# Formation of B- and M-group aflatoxins and precursors by *Aspergillus flavus* on maize and its implication for food safety

**DOI:** 10.1007/s12550-022-00452-4

**Published:** 2022-03-15

**Authors:** Alexandra Schamann, Markus Schmidt-Heydt, Rolf Geisen, Sabine E. Kulling, Sebastian T. Soukup

**Affiliations:** Department of Safety and Quality of Fruit and Vegetables, Max Rubner-Institut (MRI) - Federal Research Institute of Nutrition and Food, Karlsruhe, Germany

**Keywords:** *Aspergillus flavus*, Aflatoxins, Aspertoxin, O-Methylsterigmatocystin, Food safety

## Abstract

Aflatoxins count to the most toxic known mycotoxins and are a threat to food safety especially in regions with a warm and humid climate. Contaminated food reaches consumers globally due to international trade, leading to stringent regulatory limits of aflatoxins in food. While the formation of aflatoxin (AF) B_1_ by the filamentous fungus *Aspergillus flavus* is well investigated, less is known about the formation kinetics of its precursors and further aflatoxins. In this study, autoclaved maize kernels were inoculated with *A. flavus* and incubated at 25 °C for up to 10 days. Aflatoxins and precursors were analyzed by a validated UHPLC-MS method. Additional to AFB_1_ and AFB_2_, AFM_1_ and AFM_2_ were detected, confirming the ability of the formation of M-group aflatoxins on cereals by *A. flavus*. The measured relative levels of AFB_2_, AFM_1_, and AFM_2_ on maize compared to the level of AFB_1_ (mean of days 5, 7, and 10 of incubation) were 3.3%, 1.5%, and 0.2%, respectively. The occurrence and kinetics of the measured aflatoxins and their precursors sterigmatocystin, O-methylsterigmatocystin, 11-hydroxy-O-methylsterigmatocystin, aspertoxin, and 11-hydroxyaspertoxin (group 1) as well as of dihydrosterigmatocystin and dihydro-O-methylsterigmatocystin (group 2) supported the so far postulated biosynthetic pathway. Remarkable high levels of O-methylsterigmatocystin and aspertoxin (17.4% and 4.9% compared to AFB_1_) were found, raising the question about the toxicological relevance of these intermediates. In conclusion, based on the study results, the monitoring of O-methylsterigmatocystin and aspertoxin as well as M-group aflatoxins in food is recommended.

**Supplementary information:**

The online version contains supplementary material available at 10.1007/s12550-022-00452-4.

## Introduction

Aflatoxins (AFs) are secondary metabolites of filamentous fungi, especially produced by *Aspergillus* species like *A. flavus*, *A. minisclerotigenes*, and *A. parasiticus*, which count to the most serious fungal contaminants of food and feed (Coppock et al. 2018). AFs comprise different compounds, including aflatoxin B_1_ (AFB_1_), B_2_ (AFB_2_), G_1_ (AFG_1_), G_2_ (AFG_2_), M_1_ (AFM_1_), and M_2_ (AFM_2_) (IARC 2012). Main sources of intake of B- and G-group AFs are contaminated maize, peanuts, tree nuts, and dried fruits (Taniwaki et al. 2018). AFM_1_ and AFM_2_ have been considered until now mainly as hydroxylation products of AFB_1_ and AFB_2_, formed enzymatically in the liver of lactating dairy cows being fed with AFs contaminated feed (Min et al. 2021). Thus, AFM_1_ and AFM_2_ can especially be found as contaminants in milk and dairy products (Mohammed et al. 2016; Min et al. 2021). Little attention has been paid to limited older findings that M-/GM-group AFs can also be produced by *A. flavus* and *A. parasiticus* on/in laboratory media (Ramachandra Pai et al. 1975; Dutton et al. 1985; Yabe et al. 2012). Additionally, AFM_1_ has already been found a few times in cereals like maize and Perl millet (Matumba et al. 2015a; Abdallah et al. 2017; Houissa et al. 2019) as well as dried fruits like figs (Sulyok et al. 2020).

According to the International Agency for Research on Cancer (IARC), AFB_1_, AFG_1_, and AFM_1_ are carcinogenic with sufficient evidence in experimental animals. Limited evidence for carcinogenicity in experimental animals exists for AFB_2_ and inadequate evidence for AFG_2_. AFs in general are classified as group 1 carcinogens, due to the sufficient evidence for their carcinogenicity in humans (IARC 2012). AFB_1_, AFG_1_, and AFM_1_ are considered as pro-carcinogens. An enzymatic bioactivation by cytochrome P450 monooxygenases in the liver at the double bond at the 8,9-position in the furan ring to aflatoxin (AF)-8,9-epoxide is necessary for the carcinogenic and toxic activity (Dohnal et al. 2014; EFSA 2020). These epoxides can then form adducts with macromolecules like proteins or DNA, preferably at N7-position of the DNA guanine bases. Compared to AFB_1_, AFG_1_ has a reduced capability to intercalate into the DNA due to the less planar δ-lactone ring in its structure (Raney et al. 1990). Particularly, the AF-N7-guanine adduct formation of codon 249 of the p53 tumor suppressor gene is significant, which leads frequently to a missense mutation of this gene. The prevalence of this mutation is associated with the occurrence of hepatocellular carcinoma, as the liver is the main target tissue of AFs (Soini et al. 1996; Kucukcakan and Hayrulai-Musliu 2015).

The biosynthesis pathway of B- and G-group AFs in *A. flavus* is well elucidated. The necessary genes are grouped together in a gene cluster comprising nearly 80 kb, which is located on chromosome 3 of its genome (Yu 2012; Caceres et al. 2020). This was confirmed by whole genome sequencing of the strain used in the current study (Schamann et al. 2022). The biosynthesis pathway starts with hexanoate units from acetyl-CoA and malonyl-CoA, which are converted via intermediates to sterigmatocystin (ST) in case of the biosynthesis of AFB_1_ and to dihydrosterigmatocystin (DHST) in the biosynthesis pathway of AFB_2_. ST and DHST are then methylated to O-methylsterigmatocystin (OMST) and dihydro-OMST (DHOMST), respectively, further hydroxylated to 11-hydroxy-OMST (HOMST) and dihydro-HOMST, respectively, and then metabolized to AFB_1_ and AFB_2_, respectively, via intermediate steps (Yu 2012; Caceres et al. 2020). Additional to AFs, some of these AF precursors were investigated for their toxicological potential and revealed to have genotoxic properties (Theumer et al. 2018; Gauthier et al. 2020).

Conflicting hypotheses existed on the biosynthetic relationship between B-/G-group and M-/GM-group AFs (Biollaz et al. 1970; Dutton et al. 1985). However, this uncertainty seemed to be clarified by the postulation of a pathway of the formation of M-/GM-group AFs from OMST and DHOMST by Yabe et al. (2012). According to this, OMST and DHOMST are hydroxylated to aspertoxin (ASP) and dihydro-ASP, respectively, and further hydroxylated to 11-hydroxy-ASP (HASP) and dihydro-HASP, respectively. Both reactions are catalyzed by the enzyme OrdA. Starting from HASP and dihydro-HASP, AFM_1_ and AFM_2_ as well as AFGM_1_ and AFGM_2_ are formed via intermediates. The enzyme OrdA, which is a monooxygenase belonging to the cytochrome P450 family, is also involved in the biosynthetic pathway of AFB_1_ (Yabe et al. 2012). The postulated pathway of formation of M-/GM-group AFs would also explain the biosynthetic pathway of ASP, which has received hardly any attention compared to the other AFs so far (Benkerroum 2020). ASP was first isolated and described in 1968 (Rodricks et al. 1968a, b; Waiss et al. 1968). Adverse effects were found in developing chicken embryos, in which beak malformations, hemorrhage from umbilical vessels, edema, and loss of muscle tone were observed (LD_50_ of 0.7 µg/egg compared to the LD_50_ of 0.025 µg/egg for AFB_1_ in this study) (Rodricks et al. 1968a). Furthermore, low acute toxicity was reported in zebra fish larvae (LD_50_ of 6.6 mg/mL), showing 1/20 the acute toxicity of AFB_1_ (Abedi and Scott 1969).

The aim of the study was to elucidate the importance of M-AFs by analyzing the formation of AFs and their precursors synthesized by *A. flavus* (strain MRI19) on maize kernels as one of the most important staple foods in the world. Additionally, the formation kinetics of these compounds was investigated.

## Methods

### Reference compounds, chemicals, and reagents

The following reference standards were purchased: aflatoxin B_1_ (AFB_1_, > 99%), aflatoxin B_2_ (AFB_2_, > 99%), aflatoxin G_1_ (AFG_1_, > 99%), aflatoxin G_2_ (AFG_2_, > 99%), aflatoxin M_1_ (AFM_1_, > 98%), and sterigmatocystin (ST, > 99%) all solved in acetonitrile from Sigma-Aldrich Chemie GmbH (Taufkirchen, Germany), aflatoxin M_2_ (AFM_2_) in acetonitrile (> 98%) and aflatoxicol (AFL, > 99%) from Cfm Oskar Tropitzsch GmbH (Marktredwitz, Germany), and O-methylsterigmatocystin (OMST, > 95%) from Cayman Chemical (Ann Arbor, MI, USA). Of these standards, two standard mixtures were generated: standard mixture 1 (containing 640 nmol/L of AFB_1_, AFB_2_, AFG_1_, and AFG_2_ in acetonitrile) and standard mixture 2 (containing 640 nmol/L of AFM_1_, AFM_2_, ST, and AFL as well as 608 nmol/L of OMST in acetonitrile). All further chemicals and solvents were of analytical grade. Deionized water was obtained from an in-house ultrapure water system (LaboStar, Erlangen, Germany).

### Fungal strain and growth conditions

The strain *A. flavus* MRI19 (Schamann et al. 2022) of the culture collection of the Max Rubner-Institut was used for the experiments. The strain was originally isolated from tiger nuts, which were grown in the surrounding of Valencia in Spain. For the generation of spores, the fungus was cultivated on MG agar (malt extract [Carl Roth, Karlsruhe, Germany] 17 g/L, glucose [Carl Roth, Karlsruhe, Germany] 5 g/L, agar [Agar–Agar Kobe I; Carl Roth, Karlsruhe, Germany] 16 g/L) at 25 °C. A spore suspension was prepared using Tween-80/NaCl-mixture (NaCl [Carl Roth, Karlsruhe, Germany] 9 g/L, Tween-80 [Serva, Heidelberg, Germany] 1 g/L, agar 1 g/L). Spores were counted using a Thoma cell counting chamber (Paul Marienfeld GmbH & Co. KG, Lauda-Königshofen, Germany) and were diluted to obtain 1.0 × 10^4^ spores per mL. Autoclaved (15 min, 121 °C, 200 kPa) maize kernels (packaged popcorn maize kernels from local supermarket) were used as growth substrate. Eight grams (± 0.1 g) of maize was weighed out per petri dish (Ø = 5.5 cm) and were moistened with 1.8 mL of sterile deionized water. The maize kernels were gently stirred with the aid of a sterile pipette tip for achieving a uniform humidification. After an incubation of 24 h at 25 °C, the kernels were inoculated with 1.5 mL of the spore suspension and again stirred with a pipette tip for achieving a uniform distribution of spores. Then, the inoculated kernels were incubated for up to 10 days at 25 °C. As control, the maize kernels of eight petri dishes were neither moistened with sterile water nor inoculated with spores (control 1). Of these, the kernels of six petri dishes were used for the validation experiment of the analytical method. The kernels of further two petri dishes were moistened with 1.8 mL of sterile deionized water, and 24 h later with 1.5 mL of Tween-80/NaCl mixture without fungal spores (control 2).

### Sampling

On the day of inoculation (day 0), the samples of control 1 and 2 (see “Fungal strain and growth conditions”) were taken. Additionally, the first samples of inoculated maize kernels were taken directly after the inoculation (control 3). The further sampling was performed 1, 2, 3, 4, 5, 7, and 10 days after the inoculation. At the beginning, the empty petri dishes were marked to divide them into three equal parts. Maize kernels, which were located within one of these thirds, were determined to be one biological sample. At each sampling time, samples of six biological replicates in total were taken from two different petri dishes and were stored at −20 °C until the mycotoxin extraction.

### Mycotoxin extraction

For the mycotoxin extraction, the maize kernels were first homogenized with a ball mill (MM400, Retsch, Haan, Germany). For this, the grinding jars filled with the maize kernels of a sample and one grinding ball were pre-cooled in liquid nitrogen. Then, the kernels were homogenized for 1 min at 30 Hz. The toxin extraction was performed according to the DIN EN ISO norm 16050:2011 with some modifications (DIN 2011). For the extraction, 1 mL of methanol:water (70:30, v:v) and 40 mg of NaCl were added to 200 mg ± 2 mg of ground maize and the samples were shaken on a rotary shaker (VXR basic Vibrax®, IKA, Staufen im Breisgau, Germany) for 2 min at 2,500 rpm. The samples were centrifuged for 5 min at 16,200 × g at room temperature. The extract was transferred to a new tube and the extraction was repeated with 1 mL methanol:water (70:30, v:v). After the second centrifugation, the transferred extracts were combined. Then, the maize samples were centrifuged again for 3 min at 16,200 × g without adding further extracting agent to enable the transfer of the remains of methanol:water. The extract was vortexed and filtered through a 0.2-µm PTFE filter (Puradisc-13, Whatman™, Merck, Darmstadt, Germany). For the UHPLC-MS measurements, 100 µL of the filtrate was diluted with 100 µL of methanol:water (70:30, v:v) to receive concentrations of target analytes within the calibrated range.

### Validation of the analytical method

The analytical method for the quantitation of AFs and precursors was validated for selectivity, accuracy, precision, linearity, limit of quantitation, recovery, and matrix effect. For this, maize kernels of six petri dishes of control 1 (see “Fungal strain and growth conditions”) were homogenized as described in “Mycotoxin extraction”. Of each of these six petri dishes, one maize sample was extracted as a blank sample, following the extraction protocol described above. Additionally, of petri dish no. 1, six maize aliquots were used as pre-extract samples and further six aliquots as post-extract samples (each aliquot 200 mg ± 1 mg). For each pre-extract sample, 525 µL of standard mixtures 1 and 2 (see “Reference compounds, chemicals, and reagents”), respectively, were merged, evaporated with nitrogen, and re-dissolved in 100 µL of methanol:water (70:30, v:v). Each pre-extract sample was spiked with 100 µL of these re-dissolved standard mixtures (content of AFs in µg/kg maize: AFB_1_: 499.6, AFB_2_: 502.9, AFG_1_: 525.2, AFG_2_: 528.5, AFM_1_: 525.2, AFM_2_: 528.5, ST: 518.9, OMST: 514.2, AFL: 502.9) directly after the addition of the first milliliter of methanol:water (70:30, v:v) at the beginning of the toxin extraction. Then, the extraction protocol of the spiked samples was followed as described above. The post-extract samples were directly extracted and spiked after the sample workup. For this, instead of the final 1:1 dilution, 100 µL of the extracted samples was mixed with each 25 µL of standard mixtures 1 and 2 (containing 640 nmol/L of each target compound except for OMST [608 nmol/L]), respectively, and 50 µL of methanol:water (70:30, v:v). Additionally, six “solvent-spiked” samples were prepared. For this, 150 µL of methanol:water (70:30, v:v) was spiked with 25 µL of standard mixtures 1 and 2, respectively.

The final concentration of the target compounds in the injected pre-extract (in case of 100% recovery), post-extract as well as solvent-spiked samples was 80 nmol/L, except for OMST (76 nmol/L). The recovery of each compound was calculated by dividing the mean value of the measured peak areas of the target compounds in the pre-extract samples by the mean value in the post-extract samples. The accuracy of the measured analyte levels in the pre-extract samples was calculated by dividing the mean value of the measured concentrations of the target compounds in the pre-extract samples by the nominal concentration of 80 nmol/L (76 nmol/L for OMST) and correcting it for the individual recoveries of the compounds. The matrix effect was calculated by dividing the mean value of the measured peak areas of the target compounds in the post-extract samples by the mean value in the solvent-spiked samples. In this context, values below 100% indicate a signal suppression, whereas values above 100% a signal enhancement.

### UHPLC-MS analysis

The samples were measured on a 1290 Infinity LC system (Agilent Technologies, Waldbronn, Germany) consisting of a pump (G4220A), an autosampler (G4226A) a column oven, and a DAD (G4212A) coupled with a Triple TOF 5600 mass spectrometer (AB Sciex, Darmstadt, Germany). The separation was performed on a Waters Cortecs UPLC C18 column (2.1 mm × 150 mm, 1.6 µm; Waters, Eschborn, Germany) equipped with a pre-column (Security Guard Ultra UHPLC C18; Phenomenex, Aschaffenburg, Germany). Aqueous ammonium acetate buffer (10 mmol/L) was used as eluent A and methanol as eluent B at a flow rate of 0.25 mL per min. A gradient with the following elution profile was performed: 0.0–1.0 min isocratic with 30% B, 1.0–13.0 min from 30 to 42% B, 13.0–23.0 min from 42 to 77% B, 23.0–23.5 min from 77 to 95% B, 23.5–26.5 min isocratic with 95% B, 26.5–27.0 min from 95 to 30% B, and 27.0–37.0 min isocratic with 30% B. The column oven was set at 45 °C, and the injection volume was 1 µL. The DAD recorded from 200 to 600 nm operating with a sampling rate of 2.5 Hz. Measurements of the MS were performed in the positive ESI mode, selecting the following ionization source conditions: curtain gas 35 psi, ion spray voltage 5,500 V, ion source gas-1 50 psi, ion source gas-2 60 psi, and ion source gas-2 temperature 550 °C. The declustering potential was set to 120 V. The MS full scans were recorded from *m/z* 100–1000 with an accumulation time of 100 ms, and a collision energy voltage of 10 V. The MS/MS spectra were recorded in the high sensitivity mode from *m/z* 50–1000 with an accumulation time of 25 ms, a collision energy voltage of 35 V, and a collision energy spread of 15 V.

Analytes were identified by retention time, accurate mass, and isotope pattern. Extracted ion chromatograms (XIC) based on the accurate mass of the molecular ions of the compounds (5 mDa extraction width) were used to monitor and quantify the analytes (Table 1). For the quantitation of the target compounds, two mixtures of standard solutions (standard mixtures 1 and 2; see “Reference compounds, chemicals, and reagents”) were measured in four different concentrations (AFB_1_, AFB_2_, AFG_1_, AFG_2_, AFM_1_, AFM_2_, ST, and AFL: 640 nmol/L, 160 nmol/L, 40 nmol/L, 10 nmol/L; OMST: 608 nmol/L, 152 nmol/L, 38 nmol/L, 9.5 nmol/L) at the beginning and at the end of each measuring day to obtain a standard curve. The working standard solutions were renewed every measuring day. The standard solutions measured on the same day as the samples were used for data quantification.

**Table 1:** Analyte specific parameters of UHPLC-MS analysis, showing the retention time as well as the monitored ion species and accurate mass. Additionally, MS/MS data of analytes are displayed (precursor ion and some major product ions)

Analyte	Retention time [min]	Monitored accurate mass [Da]	Ion species	Precursor ion (MS/MS analysis) [*m/z*]	Product ions (MS/MS analysis)^a^ [*m/z*]
AFB_1_	13.2	313.07066 ± 0.00250	[M + H]^+^	313.1	285.1 (31), 284.1 (11), 270.1 (18), 269.0 (14), 241.0 (17), 214.1 (10)
AFB_2_	11.3	315.08631 ± 0.00250	[M + H]^+^	315.1	297.1 (5), 287.1 (21), 271.1 (5), 259.1 (19), 243.1 (5), 203.1 (3)
AFG_1_	9.7	329.06558 ± 0.00250	[M + H]^+^	329.1	311.1 (24), 283.1 (18), 255.1 (13), 243.1 (32), 215.1 (14), 214.1 (13)
AFG_2_	8.1	331.08123 ± 0.00250	[M + H]^+^	331.1	313.1 (16), 303.1 (6), 285.1 (7), 257.1 (6), 245.1 (9), 217.1 (4)
AFM_1_	8.5	329.06558 ± 0.00250	[M + H]^+^	329.1	301.1 (28), 273.1 (97), 259.1 (41), 258.1 (13), 255.1 (11), 229.0 (22)
AFM_2_	6.7	331.08123 ± 0.00250	[M + H]^+^	331.1	313.1 (31), 285.1 (40), 273.1 (100), 259.1 (41), 257.1 (16), 229.0 (13)
ST	22.7	325.07066 ± 0.00250	[M + H]^+^	325.1	310.0 (98), 309.0 (6), 297.1 (7), 282.1 (16), 281.0 (63), 253.0 (5)
OMST	20.7	339.08631 ± 0.00250	[M + H]^+^	339.1	324.1 (28), 311.1 (7), 306.1 (38), 295.1 (24), 278.1 (15), 277.1 (18)
HOMST	14.8	355.08123 ± 0.00250	[M + H]^+^	355.1	340.1 (2), 327.1 (20), 299.1 (54), 285.1 (30), 266.1 (20), 255.1 (12)^b^
ASP	16.4	355.08123 ± 0.00250	[M + H]^+^	355.1	340.1 (36), 327.1 (5), 322.0 (66), 294.1 (16), 293.0 (19), 266.1 (5)^b^
HASP	13.6	371.07614 ± 0.00250	[M + H]^+^	371.1	343.1 (20), 315.1 (42), 282.1 (35), 281.0 (9), 301.1 (22), 300.1 (20)^b^
DHST	22.1	327.08631 ± 0.00250	[M + H]^+^	327.1	312.1 (14), 299.1 (10), 284.1 (7), 283.1 (7), 271.1 (10), 99.0 (10)
DHOMST	19.7	341.10196 ± 0.00250	[M + H]^+^	341.1	326.1 (11), 313.1 (4), 308.1 (5), 297.1 (13), 285.1 (10), 280.1 (6)
AFL	17.3	297.07575 ± 0.00250	[M-H_2_O + H]^+^	297.1	281.1 (28), 269.1 (38), 268.1 (31), 254.1 (20), 241.1 (20), 225.1 (19)

*AFB*_*1*_ aflatoxin B_1_, *AFB*_*2*_ aflatoxin B_2_, *AFG*_*1*_ aflatoxin G_1_, *AFG*_*2*_ aflatoxin G_2_, *AFM*_*1*_ aflatoxin M_1_, *AFM*_*2*_ aflatoxin M_2_, *ST* sterigmatocystin, *OMST* O-methylsterigmatocystin, *HOMST* 11-hydroxy-O-methylsterigmatocystin, *ASP* aspertoxin, *HASP* 11-hydroxyaspertoxin, *DHST* dihydrosterigmatocystin, *DHOMST* dihydro-O-methylsterigmatocystin, *AFL* aflatoxicol

^a^Intensities of product ions are indicated in brackets in percentage

^b^Presented product ions include only ions of the revised MS/MS spectrum of the target analytes without ions of polysiloxanes

### Data analysis

For data analysis, the software MultiQuant 3.0.2 and PeakView 2.2 (AB Sciex, Darmstadt, Germany) were used. Quantification of the analytes was based on standard curves, for which linear regression with a weighting of 1/*x*^2^ was applied. Since standards were not commercially available for all target analytes, the compound which is structurally most similar to the target analyte was used for its quantification. Thus, ASP, HOMST, HASP, and DHOMST were semi-quantified based on the standard of OMST and DHST on the standard of ST. The lowest concentration of the standard curve (10 nmol/L) was set as limit of quantitation (LOQ) of all target compounds. At this concentration, the signal to noise value was 8 for AFL, between 26 and 40 for AFB_1_, AFB_2_, AFM_1_, and AFM_2_, and between 60 and 70 for AFG_1_, AFG_2_, ST, and OMST. Concentrations measured below 10 nmol/L as well as the absence of a compound in a sample were indicated as “ < LOQ” in the results. For calculating mean values, “ < LOQ” was set to 0.0 mg/kg maize. Samples containing target compounds in a higher concentration than the highest point of the standard curve (640 nmol/L) were diluted appropriately with methanol:water (70:30, v:v) to get them within the calibrated range. Then, these samples were repeatedly measured. For each target compound specifically, the dilution level, at which this compound was in the calibrated range, was used for data analysis. Statistical analyses were performed using SPSS Statistics 26 (IBM, Armonk, NY, USA).

## Results

### Mycotoxin identification

The analytes AFB_1_, AFB_2_, AFG_1_, AFG_2_, AFM_1_, AFM_2_, ST, OMST, and AFL were identified by comparison of their retention times, accurate masses in MS spectra and MS/MS spectra with those of the reference compounds. Additionally, MS/MS spectra were verified with spectra in the literature (Plattner et al. 1984; Uka et al. 2019). Furthermore, the inoculated maize samples were checked for the occurrence of the following compounds based on their accurate masses (5 mDa extraction width): AFGM_1_, AFGM_2_, ASP, HASP, HOMST, DHST, DHOMST, dihydro-ASP, dihydro-HASP, and dihydro-HOMST. Of these, a peak in the respective mass trace was detected in the inoculated maize samples for the following analytes: ASP, HASP, HOMST, DHST, and DHOMST. The MS/MS spectra of the putative ASP, DHST, and DHOMST signals (Table 1) were verified by comparison with those published by Uka et al. (2019). No MS/MS spectra were found in literature for HASP and HOMST. The measured accurate masses and isotope ratios fit to the theoretical calculated values (HASP: measured *m/z* 371.0792, +8.2 ppm mass error; HOMST: measured *m/z* 355.0811, −0.4 ppm mass error). The recorded MS/MS spectra of these two compounds were examined carefully (Table 1). In the MS/MS spectrum of the supposed HASP (hydroxylated ASP), some fragment ions with a *m/z* difference for oxygen (15.995 u) compared to fragment ions in the spectrum of ASP were recorded (e.g., *m/z* 343.083 and 282.051 compared to 327.086 and 266.056, respectively) (Table 1), which affirmed the putative identification as HASP. In the case of the supposed HOMST, an analog observation was made, namely fragment ions having a *m/z* difference for oxygen (15.995 u) to fragment ions in the spectrum of OMST (*m/z* 340.058 and 327.086 compared to 324.063 and 311.091, respectively) (Table 1).

It should be mentioned that a continuous and stable background noise of polysiloxanes (*m/z* 371.10 and 355.07) was detected. Such polysiloxane interferences were already described in a previous work (Keller et al. 2008). This polysiloxane background noise led to mixed MS/MS spectra of ASP, HOMST, and HASP, since the precursor ions in MS/MS analysis were selected by a non-high resolving quadrupole. The product ions of the MS/MS spectra of the polysiloxanes were subtracted from the mixed spectra to obtain the pure MS/MS spectra of the target analytes. Only product ions of these subtracted MS/MS spectra are listed in Table 1. However, this background noise was not relevant for the quantification of the target compounds, due to high mass resolution in MS full scan analysis.

### Validation

None of the target compounds was detected in the blank samples. For calibration curves, the best fit line was obtained by linear regression applying a weighting of 1/*x*^2^. The correlation coefficient of all analytes indicated the quality of the calibration curves and was ≥ 0.9963. Recoveries of the analytes after extraction from maize exhibited values between 85.9 and 94.3%. Thus, the measured concentrations of the analytes in the study samples were corrected for these recoveries. Matrix effect values between 97.6 and 99.1% were measured, except for AFM_2_ (93.9%) and OMST (90.0%). Recovery-corrected accuracies and intra-day precision of analytes in spiked maize were 93.0–104.1% and 0.4–3.7%, respectively. The correlation coefficients, recoveries of the extraction process, matrix effect and the precision as well as accuracies corrected for the recoveries of the pre-extract samples of the included standards are listed in Table 2.

**Table 2:** Results of the validation experiment for mycotoxin analysis in maize by UHPLC-MS with a spiking level of the injected samples of 80 nmol/L for each analyte except for OMST (76 nmol/L). The correlation coefficients (R), recoveries of the extraction process (*n* = 6), matrix effect (*n* = 6), and the intra-day precision (*n* = 6) as well as the recovery-corrected accuracies (*n* = 6) are listed

Analyte	Correlation coefficient (R)	Recovery [%]	Matrix effect [%]	Precision [%]	Accuracy^a^ [%]
AFB_1_	0.9968	89.0	98.3	2.3	104.1
AFB_2_	0.9963	88.0	99.1	3.2	103.7
AFG_1_	0.9969	88.4	97.7	1.7	101.9
AFG_2_	0.9964	89.4	98.4	0.9	102.7
AFM_1_	0.9974	90.1	98.8	1.7	100.3
AFM_2_	0.9970	90.9	93.9	2.6	95.5
ST	0.9972	85.9	97.6	1.4	99.6
OMST	0.9967	88.6	90.0	0.4	93.0
AFL	0.9970	94.3	99.0	3.7	97.4

*AFB*_*1*_ aflatoxin B_1_, *AFB*_*2*_ aflatoxin B_2_, *AFG*_*1*_ aflatoxin G_1_, *AFG*_*2*_ aflatoxin G_2_, *AFM*_*1*_ aflatoxin M_1_, *AFM*_*2*_ aflatoxin M_2_, *ST* sterigmatocystin, *OMST* O-methylsterigmatocystin, *AFL* aflatoxicol

^a^Accuracies corrected for analyte-specific recoveries

### Kinetics of AFs, their precursors, and the metabolization product AFL on maize

In our pre-experiments to this study (data not shown), an untargeted analysis of AF metabolites produced by *A. flavus* MRI19 on potato dextrose agar (PDA) was performed using UHPLC-MS. In addition to the formation of AFM_1_ (1–2% of the produced AFB_1_), high peaks of ASP and OMST were observed in this pre-experiment. The current study was performed among others to analyze, if these compounds were also produced in high levels on food like maize.

**Fig. 1:**
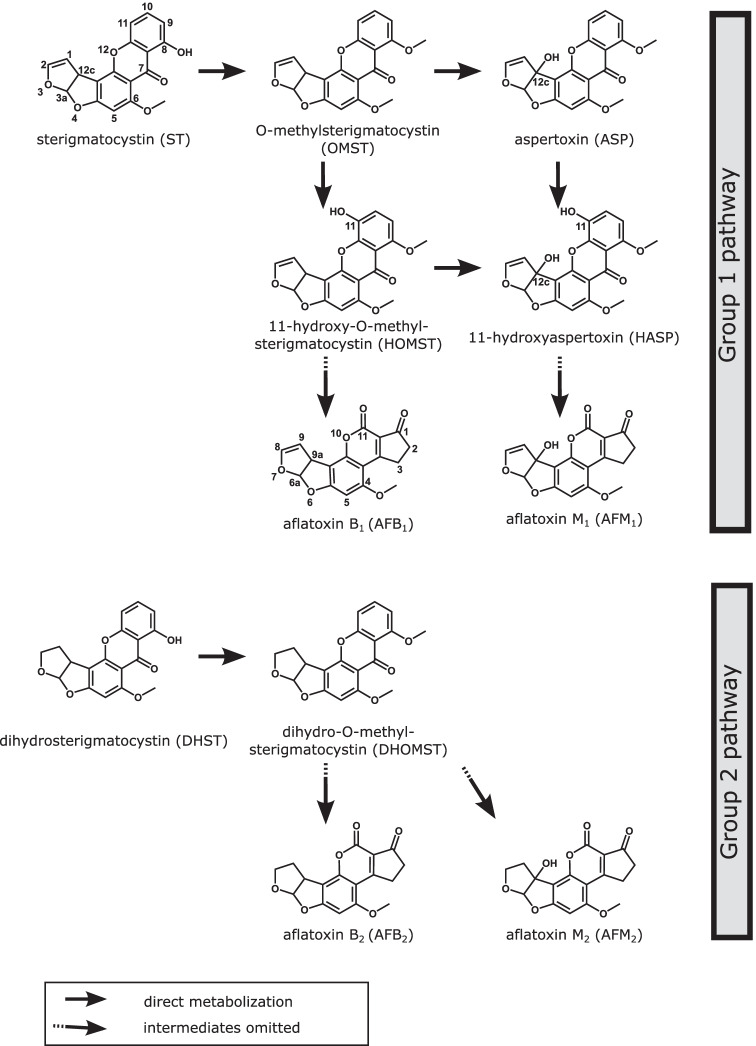
Chemical structure of aflatoxins (AFs) and precursors detected in the study. The last steps of the AF group 1 pathway for the formation of AFB_1_ and AFM_1_ and the AF group 2 pathway for the formation of AFB_2_ and AFM_2_ are shown. The pathway for the biosynthesis of B-group AFs is based on Yu (2012) and Caceres et al. (2020), and the pathway for the biosynthesis of M-group AFs is postulated by Yabe et al. (2012). The carbons were numbered regarding Pfeiffer et al. (2014)

**Fig. 2:**
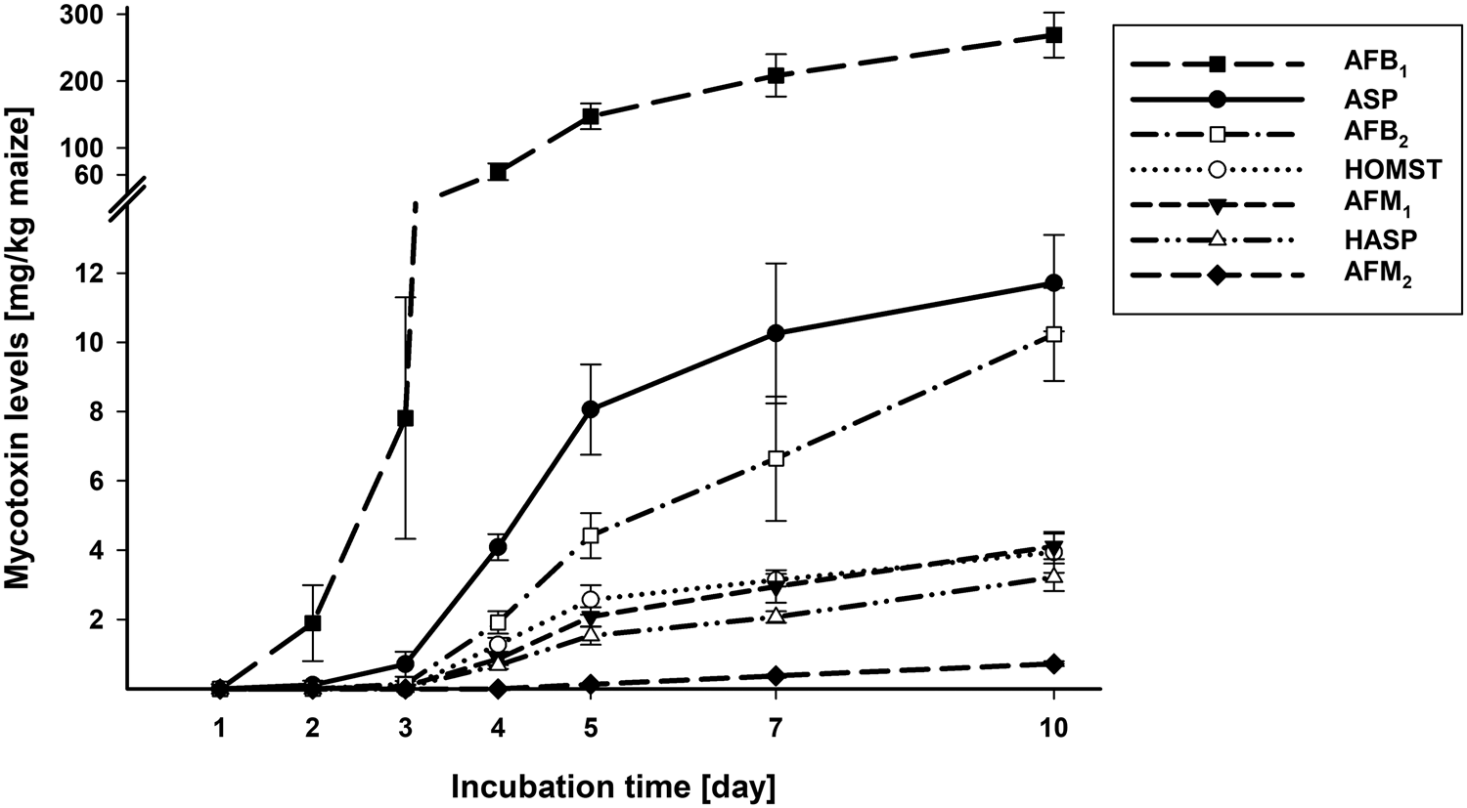
Line diagram showing the formation of aflatoxin B_1_ (AFB_1_), aflatoxin B_2_ (AFB_2_), aflatoxin M_1_ (AFM_1_), aflatoxin M_2_ (AFM_2_), aspertoxin (ASP), 11-hydroxyaspertoxin (HASP), and 11-hydroxy-O-methylsterigmatocystin (HOMST) by *A. flavus* on autoclaved maize kernels over the incubation time of 10 days. Data is given as arithmetic mean ± standard deviation of six biological samples in milligrams per kilogram of maize

**Fig. 3:**
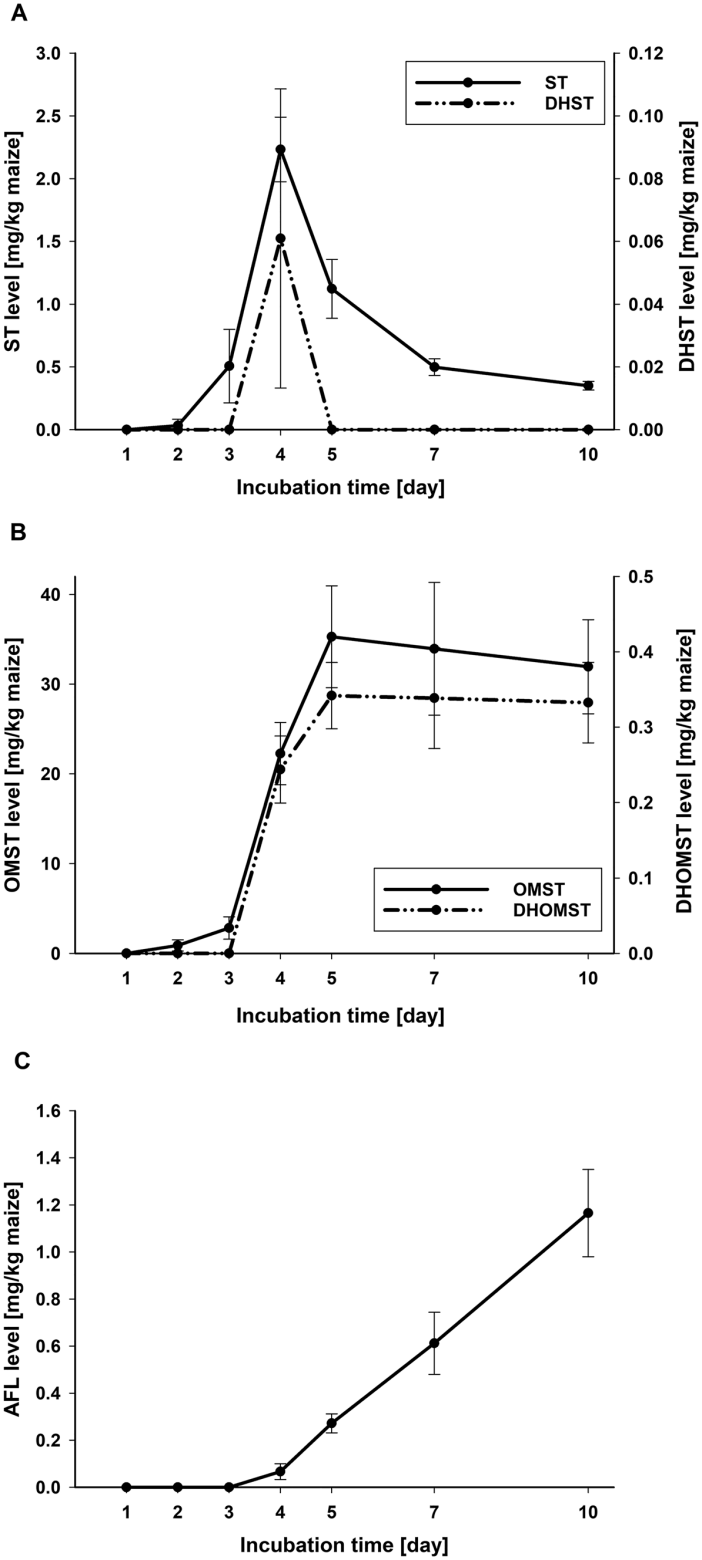
Line diagram showing the formation of the aflatoxin precursors sterigmatocystin (ST, primary *y*-axis) and dihydrosterigmatocystin (DHST, secondary *y*-axis) (**A**), as well as O-methylsterigmatocystin (OMST, primary y-axis) and dihydro-O-methylsterigmatocystin (DHOMST, secondary y-axis) (**B**), and of aflatoxicol (AFL) as metabolization product of aflatoxin B_1_ (AFB_1_) (**C**) by *A. flavus* on autoclaved maize kernels over the incubation time of 10 days. Data is given as arithmetic mean ± standard deviation of six biological samples in milligrams per kilogram of maize

**Fig. 4:**
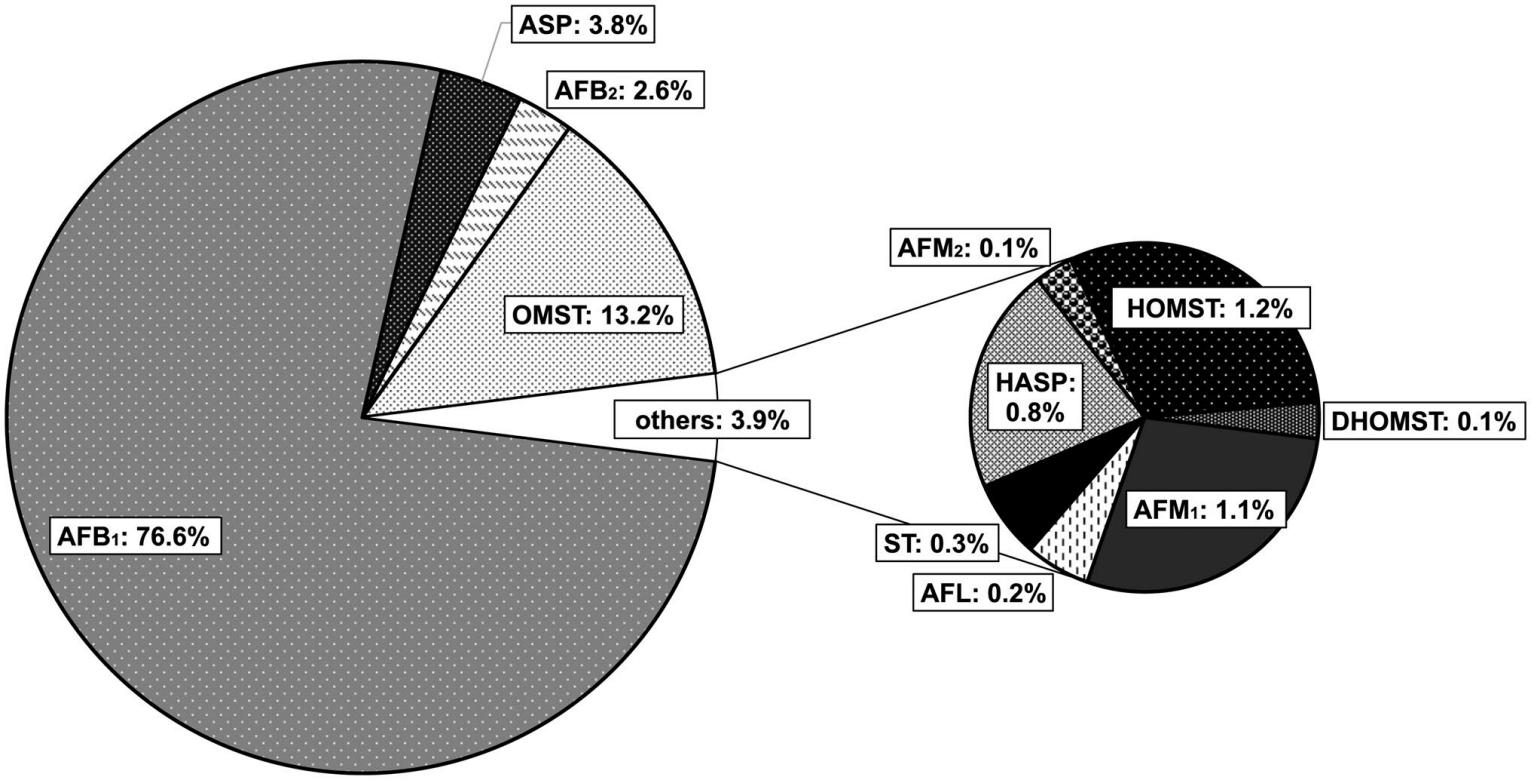
Pie chart showing the relative levels of aflatoxins and precursors related to the sum of all detected analytes presenting the arithmetic mean over days 5, 7, and 10 (*n* = 18). AFB_1_, aflatoxin B_1_; AFB_2_, aflatoxin B_2_; AFM_1_, aflatoxin M_1_; AFM_2_, aflatoxin M_2_; ASP, aspertoxin; DHOMST, dihydro-O-methylsterigmatocystin; HASP, 11-hydroxyaspertoxin; HOMST, 11-hydroxy-O-methylsterigmatocystin; ST, sterigmatocystin; OMST, O-methylsterigmatocystin; AFL, aflatoxicol

For this, autoclaved maize kernels were inoculated with a spore suspension of *A. flavus* MRI19 and were incubated for up to 10 days. The following AFs were detected in the samples: AFB_1_, AFB_2_, AFM_1_, AFM_2_, and AFL. Additionally, the samples were checked for metabolites of the last steps of the AF biosynthesis and the following precursors were detected: ST, DHST, OMST, DHOMST, HOMST, ASP, and HASP. Chemical structures of the detected compounds are shown in Fig. 1. Measured levels of AFs and their precursors on maize samples are listed in Table S1 and are illustrated in Figs. 2, 3 and 4. The Pearson correlation coefficients between selected target compounds were calculated and are listed in Table 3. In the following, the results are presented in detail for each analyte group.

**Table 3:** Pearson correlation coefficients of associations between aflatoxin/precursor levels produced by *A. flavus* on autoclaved maize kernels from days 3 to 10 of incubation

Analytes (group 1 pathway)^a^	Pearson correlation		Analytes (group 2 pathway)^b^	Pearson correlation		Analytes (corresponding metabolites)	Pearson correlation	
	*r*	*p* value		*r*	*p* value		*r*	*p* value
ST-OMST	0.046	0.810	DHST-DHOMST	0.015	0.936	ST-DHST	0.781	< 0.001*
OMST-HOMST	0.865	< 0.001*	AFB_2_-AFM_2_	0.919	< 0.001*	OMST-DHOMST	0.981	< 0.001*
OMST-ASP	0.863	< 0.001*				AFB_1_-AFB_2_	0.984	< 0.001*
HOMST-AFB_1_	0.977	< 0.001*				AFM_1_-AFM_2_	0.916	< 0.001*
ASP-HASP	0.943	< 0.001*						
HASP-AFM_1_	0.987	< 0.001*						
AFB_1_-AFM_1_	0.996	< 0.001*						

*Significant correlation (p < 0.001)

Analytes are divided as follows:

^a^Group 1 aflatoxin pathway: ST, sterigmatocystin; OMST, O-methylsterigmatocystin; HOMST, 11-hydroxy-O-methylsterigmatocystin; ASP, aspertoxin; HASP, 11-hydroxyaspertoxin; AFB_1_, aflatoxin B_1_; AFM_1_, aflatoxin M_1_

^b^Group 2 aflatoxin pathway: DHST, dihydrosterigmatocystin; DHOMST, dihydro-O-methylsterigmatocystin; AFB_2_, aflatoxin B_2_; AFM_2_, aflatoxin M_2_

#### Group 1 AFs

The first compound of the monitored pathway of group 1 AFs (AFB_1_, AFG_1_, AFM_1_) analyzed in this study is ST, which was detected in low levels from day 2 on of the incubation. A clear maximum in its formation (2.2 mg/kg maize) was detected on day 4 with a subsequent decrease to 0.35 mg ST/kg maize on day 10 (Fig. 3A, Table S1) due to its methylation to OMST. OMST was measured first on day 2 in low levels, increased strongly from days 3 to 5 and peaked on day 5 (35.3 mg/kg maize). Then, it decreased slightly until day 10 (32.0 mg/kg maize) (Fig. 3B, Table S1). OMST is hydroxylated to HOMST and, according to Yabe et al. (2012), also to ASP. Both compounds showed an increasing formation from the first quantification on day 2 for ASP and day 4 for HOMST to day 10 (Fig. 2, Table S1). From day 4, the detected level of ASP was about threefold higher than that of HOMST within the respective day. HOMST is further metabolized to AFB_1_, which was already detected after 2 days of incubation. AFB_1_ was the main metabolite of *A. flavus* in this study, being produced by far in the highest level compared to the other monitored metabolites. It showed a continuous increase over time to 269.0 mg/kg maize on day 10 (Fig. 2, Table S1). According to Yabe et al. (2012), ASP is hydroxylated to HASP and further to AFM_1_, which were both quantified the first time on day 3 and showed a very comparable, well correlated (*r* = 0.987) increase over time to 3.2 and 4.1 mg/kg maize for HASP and AFM_1_, respectively, after 10 days of incubation (Fig. 2, Tables 3 and S1). The formation of AFM_1_ correlated also very well with the AFB_1_ formation (*r* = 0.996; Table 3). AFG_1_ was not detected on maize samples inoculated with *A. flavus* MRI19 at any day of incubation.

#### Group 2 AFs

A lower number of compounds was detected of the group 2 AF pathway (AFB_2_, AFG_2_, AFM_2_). Only in a few samples DHST was measured in levels above the LOQ. DHST showed like the group 1 analog ST a peak in its formation on day 4 of incubation, after which it decreased to levels under the LOQ in all samples on days 5, 7, and 10 (Fig. 3A, Table S1). DHOMST was detected from day 4 on and increased to day 5, after which it showed a slight decrease until day 10. The kinetics of DHOMST was correlative to that of the group 1 analog OMST (Fig. 3B), showing a high association (*r* = 0.981; Table 3), although the produced level of DHOMST was only 1/100 of that of OMST. As end products of the pathway, AFB_2_ and AFM_2_ were detected. AFB_2_, which was quantified first on day 3, showed a continuous increase to 10.2 mg/kg maize on day 10 (Fig. 2, Table S1). Its kinetics was comparable to that of AFB_1_ and correlated very well (*r* = 0.984; Table 3). AFM_2_ was first measured in levels above the LOQ on day 5. Its formation increased linearly to 0.7 mg/kg maize on day 10 (Fig. 2, Table S1). AFG_2_ was not detected on maize samples inoculated with *A. flavus* MRI19 at any day of incubation.

#### Metabolization product AFL

Maize samples inoculated with *A. flavus* MRI19 were checked for AFL, a hydroxylated metabolization product of AFB_1_. AFL was detected the first time above the LOQ on day 4 of incubation and showed a continuous increase. After 10 days of incubation, 1.2 mg of AFL was measured per kilogram of maize (Fig. 3C, Table S1).

### Mycotoxin profile on maize

For the visualization of the profile of the measured AFs and their precursors on maize, the relative level of each analyte related to the sum of all target compounds was calculated. For this, the mean levels of all detected analytes (AFB_1_, AFB_2_, AFM_1_, AFM_2_, ST, DHST, OMST, DHOMST, HOMST, ASP, HASP, and AFL) were summed up separately for each day of incubation (days 5, 7, and 10). Then, the ratio between the mean produced level of each compound and the corresponding sum was calculated separately for each day (5, 7, and 10). Next, the relative levels of each analyte were averaged over days 5, 7, and 10 of incubation. The means of these three days were calculated to obtain a rather general illustration of the formation of the target compounds within the investigated incubation period, since not all compounds had their formation peak on the same day. Furthermore, the early days of incubation were omitted, since not all compounds were detected on the same day of incubation the first time. Results are shown as pie chart in Fig. 4. The highest proportions were contributed by AFB_1_ (76.6%), OMST (13.2%), and ASP (3.8%). When including only the four AFs AFB_1_, AFB_2_, AFM_1_, and AFM_2_ in this calculation, a total of 218.8 mg AFs per kilogram of maize (mean of days 5, 7, and 10) was produced, of which 95.3% belonged to AFB_1_, 3.2% to AFB_2_, 1.4% to AFM_1_, and 0.2% to AFM_2_. Since AFB_1_ is the main metabolite in this study, analog ratios related to the level of AFB_1_ instead of the sum of the levels of all analytes were further calculated. Again, the mean formation of the compounds on days 5, 7, and 10 was considered. AFB_2_, AFM_1_, and AFM_2_ were measured to 3.3%, 1.5%, and 0.2% of AFB_1_, respectively. ST and AFL were detected in low levels of 0.4% and 0.3% of AFB_1_, whereas OMST and ASP were measured in relatively high levels of 17.4% and 4.9% compared to AFB_1_.

## Discussion

The aim of the study was to analyze the formation kinetics of AFs and their precursors by *A. flavus* on maize. In general, the proposed synthesis pathway of AFs could be supported, since most of its compounds were detected. The kinetics of ST formation with a first increase and a subsequent decline in the ST level indicated a quick conversion to OMST. Thus, ST contributed quantitatively only marginally to the toxicological profile of the fungus. A rapid conversion of ST to OMST was already described by Rank et al. (2011). Yogendrarajah et al. (2015) reported low concentrations of ST compared to those of OMST and AFB_1_ as well, when analyzing the mycotoxin profile of a variety of *A. flavus* and *A. parasiticus* strains on malt extract agar by LC–MS/MS. However, other fungal species like *A. nidulans* cannot further transform ST to OMST (Brown et al. 1996; Chen et al. 2016), which could lead to relatively high ST levels in food infected by such species. In contrast to ST, OMST was detected in high levels on day 7 and 10 in the current study, which may suggest that its formation is higher than the further metabolization. Yogendrarajah et al. (2015) confirmed the formation of high levels of OMST by different strains. The measured ratios of OMST to AFB_1_ reported by Yogendrarajah et al. (2015) were mostly in a comparable range of that measured in the current study. Due to the high OMST formation, the question about its toxicological relevance raised, since it has the same structural element (double bond in the furan ring), which is responsible for the genotoxicity of AFB_1_. Little is so far known about the toxicity of OMST. When checking its genotoxicity using a hepatocyte primary culture/DNA repair test, a genotoxic effect was found due to its positive reaction for DNA repair (dose 10^−4^, 10^−5^, 10^−6^ M) (Mori et al. 1986). In contrast, no genotoxicity was observed for OMST in a recent cell study using on the one hand two human cell lines with bioactivation capabilities (HepG2 hepatoblastoma cells, LS-174 T epithelial colorectal adenocarcinoma cells) and on the other hand one human cell line with poor bioactivation capabilities (ACHN renal cell adenocarcinoma cells) (Theumer et al. 2018). However, in this study, cytotoxic effects were observed at the highest OMST concentration (100 µmol/L). Furthermore, analyzing the mutagenicity in an Ames test using *Salmonella typhimurium* with and without metabolic activation showed no mutagenic effects of OMST (0.1, 1, 10, and 100 µg/testing plate) in contrast to ST as well as AFB_1_ (Wehner et al. 1978). Thus, considering the measured levels of OMST in the current study as well as the lack of consistent data, further toxicological analyses of OMST would be necessary.

For the formation of AFB_1_, OMST is hydroxylated to HOMST by the enzyme OrdA (Udwary et al. 2002). Beyond that, the relatively low detected levels of HOMST compared to AFB_1_ (approx. 1:60) suggested that the further conversion of HOMST proceeded again relatively rapidly. Via further intermediates, which were not analyzed in this study, AFB_1_ was formed by far in the highest levels compared to all other measured metabolites. Since AF formation can be expected to increase above day 10 of incubation, the ratios of AFs and their precursors (Fig. 4) are expected to change with prolonged incubation.

The pathway of the formation of B-group AFs is well elucidated. However, less is known about the biosynthetic relationship between B-/G-group and M-/GM-group AFs. Yabe et al. (2012) postulated that M-group AFs were formed from OMST via ASP followed by HASP and further intermediates. Unexpectedly, ASP was the analyte being measured in this study in the third highest level of all analyzed metabolites. It must be noticed that no reference compound of ASP was commercially available and that ASP was quantified using the calibration curve of OMST. For this reason, the actual levels of ASP in the study samples may differ from the values reported here. Surprisingly, hardly any research was published about ASP so far, although its toxicity was already shown in developing chicken embryos in the first description of the compound (Rodricks et al. 1968a). As far as we know, this is the first report on the detection of ASP in food and on the analysis of its kinetics, which is probably due to the fact that samples were simply not tested for ASP in the past. Therefore, monitoring its presence in food is highly relevant, considering the measured levels in the current study. In addition, the toxicity of ASP needs to be investigated, as the compound (like OMST and AFB_1_) carries the toxicologically relevant double bond in the furan ring.

The end product of this branch of the postulated AF pathway is AFM_1_. In the current study, the detected level of AFM_1_ was 1.5% of that of AFB_1_. A comparable AFM_1_ formation of 1/100–1/200 of that of AFB_1_ was already described (Nakazato et al. 1991). It was suggested that the higher formation of AFB_1_ compared to AFM_1_ may be due to the higher affinity of the enzyme OrdA for the hydroxylation of OMST at the 11-carbon compared to the 12c-carbon. This would lead to the preferred formation of HOMST instead of ASP, since the same enzyme seems to be responsible for these two hydroxylations (Yabe et al. 2012). The detection of AFM_1_ in food like maize was already reported a few times. It has been speculated that insects metabolized AFB_1_ to AFM_1_ after ingesting AFB_1_ contaminated grains (Matumba et al. 2015a; Abdallah et al. 2017; Getachew et al. 2018). However, the current study demonstrated that *A. flavus* can produce AFM_1_ on maize, as a possible transformation by maize enzymes was highly likely suppressed by autoclaving the maize kernels. Furthermore, AFM_1_ was also detected in *A. flavus* cultured on PDA medium (data not shown). G-group as well as GM-group aflatoxins were not detected in the current study. This is not surprising, since *A. flavus* usually does not produce these aflatoxins in contrast to, for instance, *A. parasiticus* (Ehrlich et al. 2004).

Compared to group 1 AFs (AFB_1_, AFM_1_), low levels of group 2 AFs (AFB_2_, AFM_2_) were formed in this study, as reported in most studies analyzing AF formation (Kensler et al. 2011; Matumba et al. 2015b; Ting et al. 2020). Analogies in the kinetics of the corresponding compounds of group 1 and 2 AFs and precursors could be observed, which was already described for AFB_1_ and AFB_2_ as well as AFM_1_ and AFM_2_ (Nakazato et al. 1991).

In the current study, AFL was firstly measured two days later as AFB_1_ (day 4 vs. day 2), which suggested that AFL is a metabolization product of AFB_1_ (Detroy and Hesseltine 1970). Interestingly, a slightly different shape of formation kinetics of AFL compared to that of AFB_1_ was observed. The AFL formation seemed to start slightly exponential (days 3 to 5), followed by nearly linear formation kinetics from days 5 to 10 (Fig. 3C). In contrast, AFB_1_ formation seemed to show a slightly flattening curve from day 5 on (Fig. 2). It has to be noticed that more data points are required for a more conclusive interpretation of the shape of the curves. Although the difference between the kinetics of AFL and AFB_1_ formation is small and requires further investigation, this is in line with observations reported by Nakazota et al*.* (1991): The formation of B- and M-group AFs by most of the analyzed *A. flavus* strains investigated increased until day 15 of incubation and decreased then until day 20, whereas AFL increased beyond day 15. Furthermore, the authors described that the formation of AFB_1_ and AFL did not correlate (Nakazato et al. 1991).

In the EU, maximum levels for AFs were defined by the Commission Regulation No. 1881/2006, which was complemented by the Regulations 165/2010 and 1058/2012. Maximum levels for AFM_1_ exist only for milk, milk-based products, infant formulae, and dietary foods. Additionally, maximum levels were set for AFB_1_ as well as the sum of AFB_1_, AFB_2_, AFG_1_, and AFG_2_ for a variety of cereals, nuts, dried fruits, and spices (EU 2006, 2010, 2012). Hardly any report of the occurrence of AFM_2_ in foods other than milk and dairy products exists. This may be due to the fact that M-group AFs have so far been monitored almost exclusively in this food group. For instance, the data considered for the risk assessment of the EFSA included mainly the food categories milk and dairy products, animal and vegetable fats and oils, food for infants, and snacks, whereas M-group AFs were hardly controlled in studies analyzing for example cereals or nuts (EFSA 2020). However, since the current study clearly demonstrates that AFM_1_ and AFM_2_ can occur in food beside milk and dairy products, the monitoring of these AFs would be necessary in the same food categories regularly monitored for B- and G-group AFs. If it was confirmed that M-group AFs were frequently found in these food categories as well, it would be necessary to discuss, whether M-group AFs should be included in the EU sum maximum level of AFs (currently sum of AFB_1_, AFB_2_, AFG_1_, and AFG_2_) for different foodstuffs. The inclusion of M-group aflatoxins in this sum level should be considered, especially when keeping in mind that the carcinogenicity of AFM_1_ is better confirmed than that of AFB_2_ and AFG_2_ (IARC 2012). For AFM_2_, the toxicity is still relatively unknown and, thus, it was not included in the risk assessment of AFs in food of the EFSA (EFSA 2020). However, we support the opinion of the EFSA that further data on AFM_2_ are needed (EFSA 2020). Beside the evaluation of M-group AFs, it should be considered in further studies, in how far toxicologically relevant precursors, like versicolorin A, contribute to health risk. After a subsequent risk assessment, it would be necessary to examine, whether such precursors must also be regulated in food.

To the best of our knowledge, this study is the first detailed description of the kinetics of precursors of AFs on food (maize kernels). However, further toxicological relevant precursors of AFs are known (e.g., versicolorin A) (Theumer et al. 2018; Gauthier et al. 2020), which were not monitored in this study, since a valid semi-quantification of these compounds was not fully achievable due to lack of the (structural related) reference standards. Further studies should be conducted to elucidate the quantitative relevance of these toxins in food. Due to the laborious sample preparation, the application of a validated method, and the inclusion of six biological replicates per sampling day, it was possible to show the biological variation rather than the technical variation. This can be demonstrated by the relatively low standard deviations. Unfortunately, standards were not commercially available for all analyzed compounds. Thus, it was necessary to estimate the concentration of those compounds based on the available standards of closely related analytes. However, this has no influence on the shape of the kinetics of these compounds. Furthermore, it should be mentioned that autoclaved maize kernels were used, which might alter the maize surface structure and might facilitate the infection of the fungus compared to unprocessed maize. Additionally, this did not fully represent the common situation of stored maize. However, the autoclavation of the maize kernels was necessary to inactivate other microorganisms and to suppress the activity of maize enzymes.

In conclusion, the study showed the formation of B- and M-group AFs and precursors like ST, OMST, and ASP on maize being produced by *A. flavus*. The kinetics of the detected compounds was described in detail in the present study. The results indicate that these compounds could possibly be found in contaminated food in relevant levels. Therefore, the monitoring of the occurrence of M-group AFs, ASP, and OMST in food as well as further investigation of the toxicological potency of ASP and OMST are suggested in order to enable and/or improve a risk assessment of these compounds.

## Supplementary information

Below is the link to the electronic supplementary material.

Supplementary file1 (PDF 19 KB)

## Abbreviations

AF: Aflatoxin
AFB_1_: Aflatoxin B_1_
AFB_2_: Aflatoxin B_2_
AFG_1_: Aflatoxin G_1_
AFG_2_: Aflatoxin G_2_
AFM_1_: Aflatoxin M_1_
AFM_2_: Aflatoxin M_2_
ST: Sterigmatocystin
OMST: O-Methylsterigmatocystin
HOMST: 11-Hydroxy-O-methylsterigmatocystin
ASP: Aspertoxin
HASP: 11-Hydroxyaspertoxin
DHST: Dihydrosterigmatocystin
DHOMST: Dihydro-O-methylsterigmatocystin
AFL: Aflatoxicol
LOQ: Limit of quantitation
