# Supplementary material for: Formation of B- and M-group aflatoxins and precursors by Aspergillus flavus on maize and its implication for food safety
Source: Mycotoxin Res. 2022 Mar 15;38(2):79–92. doi: 10.1007/s12550-022-00452-4 (PMC9038868; doi:10.1007/s12550-022-00452-4)
Supplement: Supplementary file 1 — Supplementary file1 (PDF 19 KB) [file 12550_2022_452_MOESM1_ESM.pdf]

Supplementary Information

Formation of B- and M-group aflatoxins and precursors by *Aspergillus flavus* on maize and its implication for food safety

Alexandra Schamann, Markus Schmidt-Heydt, Rolf Geisen, Sabine E. Kulling and Sebastian T. Soukup*

Max Rubner-Institut (MRI) - Federal Research Institute of Nutrition and Food, Department of Safety and Quality of Fruit and Vegetables, Karlsruhe, Germany

* corresponding author: Sebastian.Soukup@mri.bund.de

Table S1 Measured levels of aflatoxins and their precursors on autoclaved maize kernels produced by *A. flavus* after 1, 2, 3, 4, 5, 7 and 10 days of incubation. Data are given as arithmetic mean ± standard deviation (n = 6) in mg/kg maize. Analytes are sorted as follows: group 1 aflatoxin pathway: ST, sterigmatocystin; OMST, O-methylsterigmatocystin; HOMST, 11-hydroxy-O-methylsterigmatocystin; ASP, aspertoxin; HASP, 11-hydroxyaspertoxin; AFB_1, aflatoxin B_1; AFM_1, aflatoxin M_1; group 2 aflatoxin pathway: DHST, dihydrosterigmatocystin; DHOMST, dihydro-O-methylsterigmatocystin; AFB_2, aflatoxin B_2; AFM_2, aflatoxin M_2; metabolization product: AFL, aflatoxicol.

Incubation time [day]	1	2	3	4	5	7	10
ST	< LOQ[a]	0.03 ± 0.05	0.51 ± 0.29	2.23 ± 0.26	1.12 ± 0.23	0.50 ± 0.07	0.35 ± 0.03
OMST	< LOQ	0.89 ± 0.62	2.82 ± 1.24	22.25 ± 3.46	35.29 ± 5.69	33.94 ± 7.40	31.95 ± 5.25
HOMST[b]	< LOQ	< LOQ	< LOQ	1.27 ± 0.20	2.57 ± 0.42	3.14 ± 0.18	3.94 ± 0.59
ASP[b]	< LOQ	0.11 ± 0.12	0.71 ± 0.36	4.09 ± 0.37	8.06 ± 1.31	10.26 ± 2.02	11.71 ± 1.39
HASP[b]	< LOQ	< LOQ	0.04 ± 0.06	0.68 ± 0.11	1.53 ± 0.26	2.04 ± 0.12	3.22 ± 0.40
AFB_1	< LOQ	1.90 ± 1.09	7.81 ± 3.48	64.36 ± 12.42	147.28 ± 19.16	208.60 ± 31.69	268.97 ± 33.70
AFM_1	< LOQ	< LOQ	0.07 ± 0.06	0.86 ± 0.18	2.08 ± 0.28	2.95 ± 0.47	4.11 ± 0.37
DHST[b]	< LOQ	< LOQ	0.19 ± 0.47	0.06 ± 0.05	< LOQ	< LOQ	< LOQ
DHOMST[b]	< LOQ	< LOQ	< LOQ	0.24 ± 0.04	0.35 ± 0.05	0.34 ± 0.07	0.33 ± 0.05
AFB_2	< LOQ	< LOQ	0.13 ± 0.09	1.91 ± 0.32	4.42 ± 0.65	6.64 ± 1.80	10.23 ± 1.34
AFM_2	< LOQ	< LOQ	< LOQ	< LOQ	0.13 ± 0.01	0.37 ± 0.05	0.72 ± 0.07
AFL	< LOQ	< LOQ	< LOQ	0.07 ± 0.03	0.27 ± 0.04	0.61 ± 0.13	1.16 ± 0.19

[a] < LOQ (limit of quantitation): levels in all replicates were below LOQ (10 nmol/L in the injected sample; in mg/kg maize: ST: 0.06; OMST: 0.07; HOMST: 0.07; ASP: 0.07; HASP: 0.07; AFB_1: 0.06, AFM_1: 0.07; DHST: 0.07; DHOMST: 0.07; AFB_2: 0.06; AFM_2: 0.07; AFL: 0.06)
[b] Analyte was semi-quantified using the structurally most similar available compound (ST or OMST)
